# Robustness of transcriptional regulatory program influences gene expression variability

**DOI:** 10.1186/1471-2164-10-573

**Published:** 2009-12-02

**Authors:** Zhiming Dai, Xianhua Dai, Qian Xiang, Jihua Feng

**Affiliations:** 1 Electronic Department, Sun Yat-Sen University, Guangzhou, PR China

## Abstract

**Background:**

Most genes are not affected when any transcription factor (TF) is knocked out, indicating that they have robust transcriptional regulatory program. Yet the mechanism underlying robust transcriptional regulatory program is less clear.

**Results:**

Here, we studied the cause and effect of robust transcriptional regulatory program. We found that cooperative TFs in the robust transcriptional regulatory program regulate their common target genes in an activity-redundant fashion, and they are able to compensate for each other's loss. As a result, their target genes are insensitive to their single perturbation. We next revealed that the degree of robustness of transcriptional regulatory program influences gene expression variability. Genes with fragile (unrobust) transcriptional regulatory program under normal growth condition could be readily reprogrammed to significantly modulate gene expression upon changing conditions. They also have high evolutionary rates of gene expression. We further showed that the fragile transcriptional regulatory program is a major source of expression variability.

**Conclusion:**

We showed that activity-redundant TFs guarantee the robustness of transcriptional regulatory programs, and the fragility of transcriptional regulatory program plays a major role in gene expression variability. These findings reveal the mechanisms underlying robust transcription and expression variability.

## Background

Proper control of gene expression is critical for the complex function of a living cell. Although gene expression can be regulated at multiple levels, one of the most important regulatory mechanisms is at the transcriptional level. The transcriptional program is regulated by binding of transcription factors (TFs) to the specific DNA sequences in regulatory regions of the genes. TFs are thus fundamental to the regulation of gene expression. However, several studies on specific TFs have observed that only a small fraction of genes that were bound by a TF were differentially expressed after that factor was knocked out or knocked down [1-4]. Recently, a genome-wide study has carried out knockout experiments to determine knockout target genes (i.e. differentially expressed genes) for 269 budding yeast TFs under normal growth condition [5]. Further analysis showed that there is only a low overlap (~3%) between the knockout targets and the binding targets identified by chromatin immunoprecipitation (ChIP)-chip [6].

There are several possible explanations for the small overlap between knockout and binding targets. First, the large-scale ChIP-chip data set could be more likely to be affected by experimental noise [5]. The overlap between Rap1 knockout and binding targets from a different high-quality ChIP-chip data set [7] is as twice as that for the large-scale ChIP-chip data set [6]. Second, binding and knockout experiments could capture distinct features of the transcriptional regulatory program [8]. Binding experiments show a preference to uncover long-range interactions between telomeres and transcription factors, while knockout experiments tend to reveal downstream effects of interference with ribosome biogenesis. Third, mere TF binding is not sufficient to guarantee its regulation. The effect of bound TFs on target genes' expression might depend on the presence of other proteins. The location, orientation and spacing of transcription factor binding sites (TFBSs) also affect the regulatory function of TFs on their target genes [9,10]. Finally, the knockout targets could include both direct and indirect targets. The overlap between the knockout and the binding targets indeed significantly improved after the elimination of the indirect targets [5].

The small overlap between knockout and binding targets might be indicative of redundant TFs which mask the TF knockout effect. Most eukaryotic genes are regulated by a combination of TFs [11-13]. Some TFs might work in a redundant fashion [14], and they could compensate for each other's loss [15,16]. The compensation among TFs should lead to the insensitivity of binding targets to the knockout of single TF. We referred to this insensitivity to the knockout of single TF as the robustness of transcriptional regulatory program. An interesting question arises concerning how the TFs involved in robust transcriptional regulatory program work in a redundant fashion. In addition, it is intuitive that this robustness should influence gene expression, but evidence for the effects remains to be elucidated.

In this study, we first distinguished between robust and fragile (unrobust) transcriptional regulatory programs according to their degrees of sensitivity to the knockout of TFs under normal growth condition. We referred to TFs involved in robust or fragile transcriptional regulatory program as robust or fragile TFs, and referred to genes having robust or fragile transcriptional regulatory program as robust or fragile genes. We found that robust cooperative TFs show significantly higher co-activity than fragile cooperative TFs. This result indicates that robust TFs regulate their common target genes in an activity-redundant fashion. We further revealed that fragile genes have high capacity to modulate gene expression upon changing conditions and have high evolutionary rates of genes expression. Paralogs provide backup mechanisms for expression variability of fragile genes. We compared the fragility of transcriptional regulatory program with other determinants of expression variability, and showed that the fragility is an important source of expression variability.

## Results

### Identification of robust and fragile TFs

We first identified robust and fragile TFs by using genome-wide TF binding data [6], TF knockout data [5] and gene expression data [17-19]. For the gene expression data sets [17-19], we refined the data measured under normal growth condition, the condition at which the TF binding and knockout data were generated. If a given TF is involved in the robust transcriptional regulatory program, the expression of its binding target genes (i.e. the TF cohort) should not be affected by any TF knockouts. However, some TFs might merely bind promoters without any regulatory function, and their single knockout should not affect the expression of their cohort genes regardless of robust or fragile transcriptional regulatory program. We should identify these TFs and exclude them for analysis. If the TF regulates expression of its cohort genes, the pair-wise Pearson correlation coefficients among expression profiles of its cohort genes should be significantly higher than genome-wide level. Accordingly, we focused our analysis on TFs whose cohort genes have significantly higher pair-wise Pearson correlation coefficients in expression profiles. If a given TF is involved in the fragile transcriptional regulatory program, the expression of its cohort genes should be significantly affected by its knockout. We used the Kolmogorov-Smirnov (K-S) statistical test to measure the discrepancy in the distribution of TF-knockout gene expression values between a given TF cohort and the rest of the genes. Our test generated 70 robust TFs whose cohort genes are not differentially expressed upon any TF knockouts (*P *> 0.05, K-S test), and 13 fragile TFs whose cohort genes are differentially expressed upon their knockouts (Bonferroni corrected *P *< 0.01, K-S test; see Materials and Methods) (Additional file 1). The big difference in the numbers of robust and fragile TFs is consistent with the small overlap between knockout and binding targets [5]. In addition, the robust cohort genes are also less sensitive to the changes in expression of their associated TFs: robust TFs are significantly less co-expressed with their cohort genes than fragile TFs (*P *< 10^-35^, Mann-Whitney U-test; Additional file 2). Together, these results show that our identified robust cohort genes are relatively insensitive to the perturbation of their associated TFs.

As transcription regulation in most eukaryotic genes is not controlled by a single TF but by multiple TFs, we next identified cooperative TFs for each robust or fragile TF. Given a TF *A*, if another TF *B *works together with *A*, the *A *cohort should be significantly enriched with the *B *binding target genes. We used the Mann-Whitney U-test to evaluate the difference in the medians of experimentally measured *B *binding affinities between the *A *cohort and the rest of the genes. 62 out of 70 robust TFs, and 12 out of 13 fragile TFs have at least one cooperative TF (Additional file 1), that is, the binding affinities of the TF cohort genes are significantly higher than those of the other genes for at least one other TF (Bonferroni corrected *P *< 0.01, Mann-Whitney U-test). In addition, there is no significant difference in the number of cooperative TFs between robust and fragile TFs (*P *= 0.95, Mann-Whitney U-test).

### Robust cooperative TFs show high co-activity

**Figure 1:**
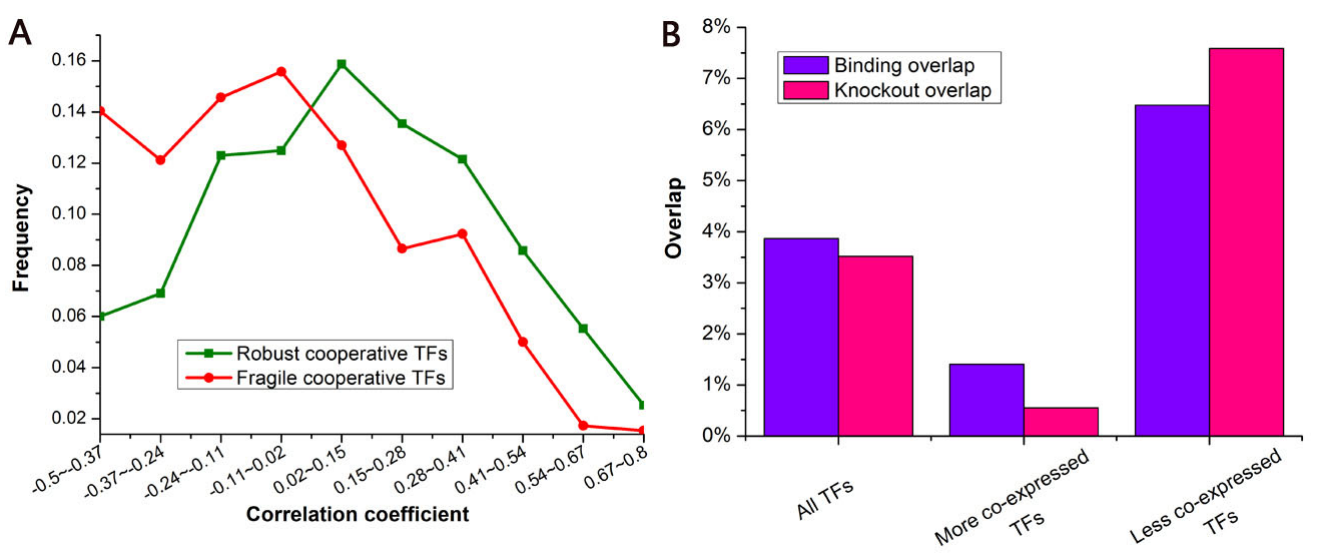
**The difference between robust and fragile transcriptional regulatory programs in TF co-activity**. (A) We calculated pair-wise Pearson correlation coefficient in expression profiles between robust cooperative TFs or fragile cooperative TFs. Distributions of resulting correlation coefficient values are presented for robust cooperative TFs (green) and fragile cooperative TFs (red). Higher positive correlation indicates high co-activity (co-expressed). Robust cooperative TFs show higher co-activity than fragile cooperative TFs. (B) The binding and knockout overlap for all TFs and two subsets of TFs (see Materials and Methods). TFs that are more co-expressed (co-activity) with all other TFs have lower overlap, while TFs that are less co-expressed with all other TFs have greater overlap. Low overlap indicates robust transcriptional regulatory program.

We next asked how cooperative TFs contribute to the robustness of transcriptional regulatory program. Cooperative TFs work in concert to regulate a set of genes. In the robust transcriptional regulatory program, cooperative TFs could compensate for each other's loss. We speculated that there might be redundancy in TF activity level in the robust transcriptional regulatory program. Like the method in a previous study [20], we used gene expression profile as a close approximation to activity level. Using gene expression profiles under normal growth condition [17-19], we found that cooperative TFs in the robust transcriptional regulatory program are significantly more co-expressed than those in the fragile program (*P *< 10^-18^, Mann-Whitney U-test; Figure 1A). Conversely, we examined whether TFs that are more co-expressed with the other TFs tend to be involved in the robust transcriptional regulatory program. We found that TFs that are significantly more co-expressed with the other TFs show a fourfold decrease in the overlap between their knockout and binding targets compared with TFs that are significantly less co-expressed with the other TFs (Figure 1B). A low overlap between knockout and binding targets for a given TF reflects the high insensitivity of TF binding targets to the TF knockout (i.e. robust transcriptional regulatory program). While this manuscript was in preparation, a study has revealed that TFs with more similar TF paralogs had lower overlap between their binding and knockout targets [21]. Our identified TFs that are significantly more co-expressed with the other TFs show a low overlap with the TFs having the most similar TF paralogs identified in that study (hyper-geometric *P *= 0.07). This result suggests that TF paralogs and TF co-activity (i.e. co-expression) are different mechanisms underlying the robust transcriptional regulatory program.

### Fragile genes have high transcriptional plasticity

We have analyzed robust and fragile transcriptional regulatory programs in terms of their TFs, and next would turn our analysis to robust or fragile genes (i.e. genes with robust or fragile transcriptional regulatory program). Using the refined transcriptional regulatory network without indirect regulatory interactions constructed from the TF knockout data [5], we denoted the transcriptional regulatory program as '*k *fragility (the degree of fragility)' to a specific gene according to the number *k *of TF knockouts which it is affected by. As most genes (~60%) are 0-fragile genes, we focused our analysis on the genes that are affected by the knockout of a large number of TFs. On average, genes were affected by the knockout of two TFs. We determined genes as fragile genes if they are 5-fragile or higher. We identified a total of 785 fragile genes (Additional file 3). A recent study has measured genome-wide gene expression levels on overexpression of 55 TFs [22]. Fragile genes are sensitive not only to TF knockout but also to TF overexpression: their expression levels are significantly more changed upon TF overexpression than the rest of the genes (*P *< 10^-35^, Mann-Whitney U-test; Additional file 4).

**Figure 2:**
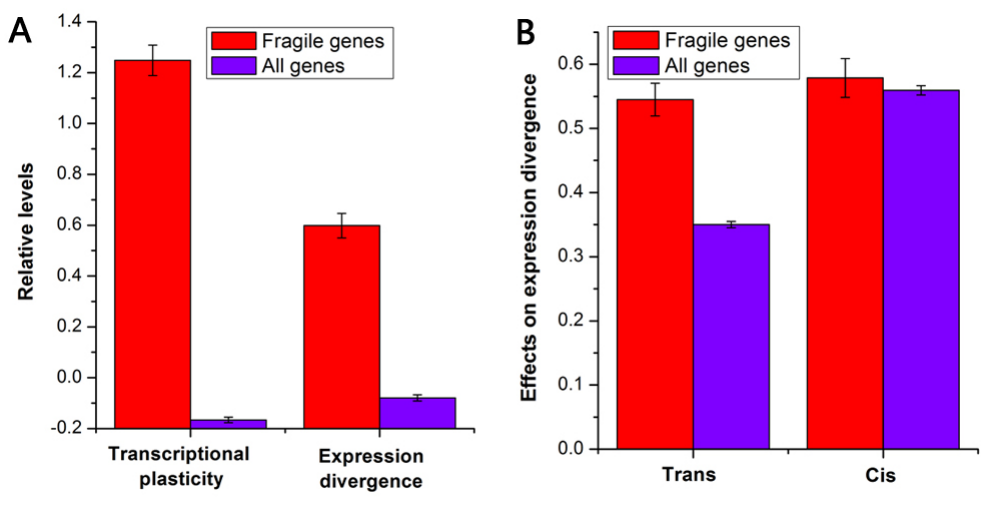
**Features in gene expression that are specific for fragile genes**. (A) Average values that correspond to transcriptional plasticity and expression divergence between yeast species are shown for fragile genes (red) and all genes (violet). Values in each property were normalized, such that their means are zero and standard deviations are one. (B) Average values that correspond to *trans *and *cis *effects on expression divergence are shown for fragile genes (red) and all genes (violet). Error bars were calculated by bootstrapping.

We next examined how fragile genes modulate gene expression upon changing environmental conditions. As fragile genes have transcriptional regulatory programs that are sensitive to TF perturbation under normal growth condition, an open question is whether this property makes their expression more readily be reprogrammed in response to changing conditions. To test this possibility, we used gene expression data from the Stanford Microarray Database [23] to calculate for each gene the average magnitude of expression changes upon various conditions (see Materials and Methods), termed as transcriptional plasticity. The transcriptional plasticity quantifies the dynamic range of expression level in various conditions. Fragile genes have significantly higher transcriptional plasticity than the rest of the genes (*P *< 10^-175^, Mann-Whitney U-test; Figure 2A). This result also persisted when transcriptional plasticity was calculated separately for up-regulation or down-regulation (Additional file 5). These results indicate that fragile genes show more expression changes under a variety of conditions regardless of the direction of the expression changes. Furthermore, transcriptional plasticity globally increases with the degree of fragility (*R *= 0.43, *P *≈ 0; Additional file 6).

We further investigated into the relationship between fragile transcriptional regulatory program under normal growth condition and gene expression responses to different stress conditions. It is well established that yeast cells regulate expression levels of ~900 genes in a stereotypical manner in most of the environmental stress conditions, commonly referred to as the environmental stress response (ESR) [19]. TFs bind some specific genes to regulate their expression in response to changing conditions. Using the genome-wide TF binding data measured in various conditions [6], we identified genes (*n *= 631;Additional file 7) that are bound by multiple (at least two) TFs in the stress conditions that were included in ESR [19]. These multiple-TF-binding genes were expected to be regulated in response to environmental stress conditions. However, only a small fraction (~17%) of these genes participate in ESR. We found that these minority genes are affected by more TF knockouts under normal growth condition than the rest of multiple-TF-binding genes (5.4 versus 2.7; *P *< 10^-12^, Mann-Whitney U-test). This result indicates that these minority genes have relatively fragile transcription regulatory program under normal growth condition. Moreover, these two gene classes show no significant difference in TF binding number in stress conditions (2.6 versus 3.0), ruling out the possibility that their difference in environmental response is attributable to their difference in TF binding number. Together, these results suggest that fragile transcriptional regulatory program under normal growth condition tend to be reprogrammed in response to changing conditions.

**Figure 3:**
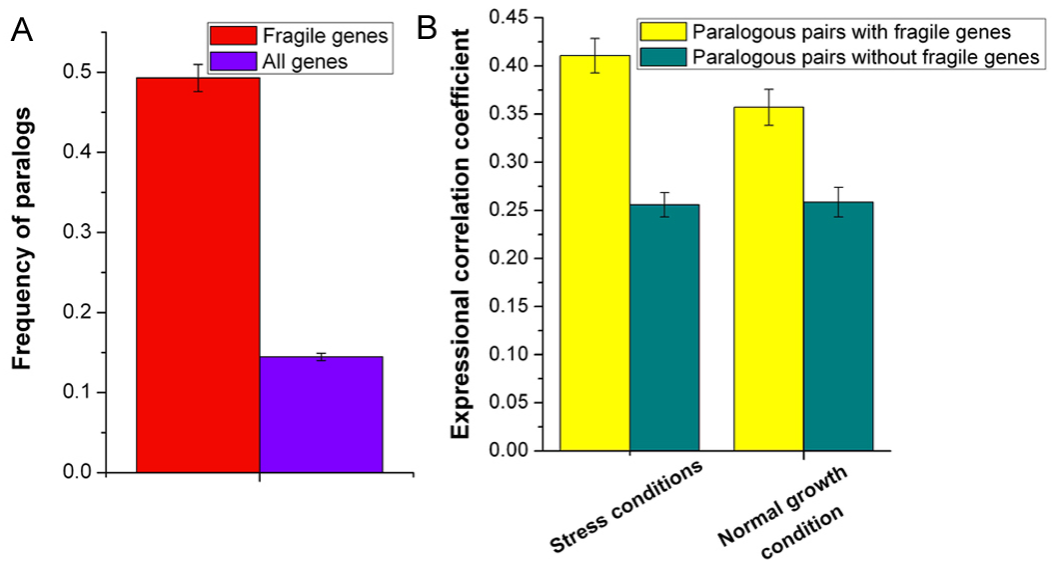
**Backup mechanisms used for fragile genes**. (A) Frequency of paralogs is shown for fragile genes (red) and all genes (violet). (B) Pair-wise Pearson correlation coefficient in expression profiles was calculated for each paralogous gene pairs under both stress and normal growth conditions. Average values that correspond to expressional correlation coefficient are shown for paralogous pairs with fragile genes (yellow) and paralogous pairs without fragile genes (dark cyan). Error bars were calculated by bootstrapping.

We next examined whether there are other mechanisms that ensure normal expression of fragile genes in various conditions. A previous study has identified 1,269 paralogs in yeast [24]. Fragile genes are enriched with paralogs (Figure 3A), which indicates backup mechanisms might be used for fragile genes. Indeed, the paralogous gene pairs involving fragile genes are more pair-wise co-expressed than the other paralogous gene pairs under both normal growth and stress conditions (*P *< 10^-16 ^for stress conditions, *P *< 10^-8 ^for normal growth conditions, Mann-Whitney U-test; Figure 3B). These results suggest that paralogs provide backup mechanisms and robustness for expression of fragile genes in various conditions. Fragile genes have high evolutionary rates of genes expressionWe next asked whether fragile transcriptional regulatory program is linked to high evolutionary rate of gene expression. Fragile genes have higher evolutionary rates of gene expression between yeast species [25] than the rest of the genes (*P *< 10^-50^, Mann-Whitney U-test; Figure 2A). Evolution of gene expression can be caused by changes in *cis *and/or *trans *regulations. Previous studies have experimentally quantified the relative contribution of *cis *and *trans *effects to expression divergence between species, and have revealed that most of interspecies divergence can be generally explained by *cis *effects [26,27]. However, *trans *and *cis *effects [27] of fragile genes show comparable contribution to expression divergence between species (*P *= 0.27, Mann-Whitney U-test). Fragile genes have higher *trans *effects than the rest of the genes (*P *< 10^-9^, Mann-Whitney U-test; Figure 2B), and have comparable *cis *effects with the other genes (*P *= 0.68, Mann-Whitney U-test). Hence, fragile transcriptional regulatory program might facilitate the contribution of *trans *effects on expression divergence.

Sensory *trans *effects are suggested to play a dominant role in *trans *effects [27]. Accordingly, ESR genes show higher *trans *effects. We sought to understand the contribution of sensory *trans *effects and fragile transcriptional regulatory program to *trans *effects. To this end, we compared *trans *effects between fragile and ESR genes. We excluded genes shared by these two gene classes for analysis. These two gene classes show comparable *trans *effects and evolutionary rates of gene expression (*P *= 0.42 and *P *= 0.57, Mann-Whitney U-test).

### Fragile transcriptional regulatory program is a major source of expression variability

**Figure 4:**
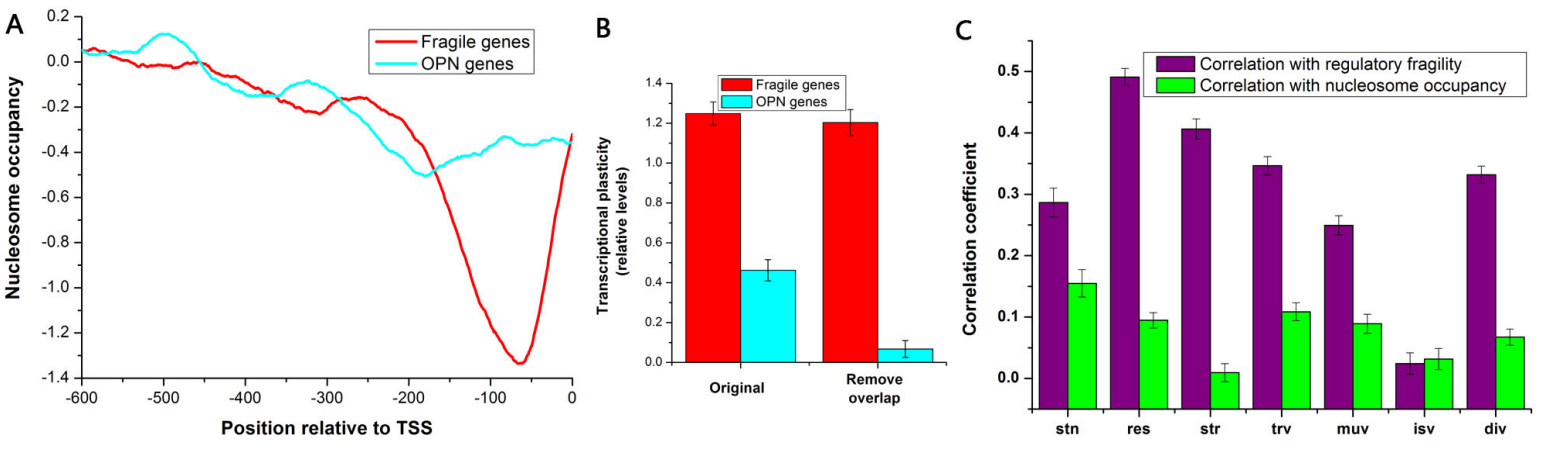
**Fragile transcriptional regulatory program is a major source of expression variability**. (A) Average nucleosome profiles in promoter regions are shown for fragile genes (red) and OPN genes (cyan). OPN genes have higher nucleosome occupancy close to the TSS. (B) Comparison of transcriptional plasticity was performed between fragile genes (red) and OPN genes (cyan). Values were normalized, such that their means are zero and standard deviations are one. When removing the genes that are shared by fragile genes and OPN genes, the difference became more significant and OPN genes have transcriptional plasticity comparable to genome-wide level. (C) Multiple regression analysis of two properties influencing seven measures for expression variability, including stochastic noise (stn), responsiveness (res), stress response (str), trans variability (trv), mutational variance (muv), interstrain variation (isv) and expression divergence (div). The effects of fragile transcriptional regulatory program (purple) and the presence of nucleosomes close to the TSS (green) on expression variability were estimated by multiple linear regression of the two properties. Error bars in B and C were calculated by bootstrapping.

We next sought to quantify the contribution of fragile transcriptional regulatory program to expression variability. Expression variability is primarily regulated at the chromatin level [28]. Nucleosome occupancy at the 150 bp upstream of the transcription start site (TSS) shows high positive correlation with transcriptional plasticity [29]. 544 occupied proximal-nucleosome (OPN) genes with high nucleosome occupancy close to the TSS were identified [29]. Fragile genes have significantly lower nucleosome occupancy [30] close to the TSS [31] than OPN genes (Figure 4A), indicating that fragile transcriptional regulatory program has an impact on expression variability irrespective of the presence of nucleosomes close to the TSS. We found that fragile genes have higher transcriptional plasticity than OPN genes (*P *< 10^-24^, Mann-Whitney U-test; Figure 4B). When excluding the genes shared by fragile and OPN genes, the difference became more significant (*P *< 10^-35^, Mann-Whitney U-test) and OPN genes have transcriptional plasticity comparable to genome-wide level (Figure 4B). Although transcriptional plasticity is also linked to the presence of a TATA box [24], we found that fragile genes have still higher transcriptional plasticity than TATA-containing genes [32] (Additional file 8). To further investigate into the contribution of fragile transcriptional regulatory program and chromatin structure to expression variability, we used seven measures for expression variability as in a previous study [33], including stochastic noise [34], responsiveness [25], stress response [19], *trans *variability [28], mutational variance [35], interstrain variation [36] and expression divergence [25]. We used multiple linear regression to analyze the correlations of the seven variability measures with the fragility of transcriptional regulatory program and the average nucleosome occupancy within the 150 bp upstream of the TSS (Figure 4C). We found that the fragility of transcriptional regulatory program is more positively correlated with six out of seven variability measures than nucleosome occupancy. The two factors show comparable correlation with the other variability measure (interstrain variation). We conclude that fragile transcriptional regulatory program plays a more fundamental role in expression variability compared with the presence of nucleosomes close to the TSS.

## Discussion

We have shown that TF redundancy in activity plays an important role in the robustness of transcriptional regulatory program. In addition, TF redundancy in sequence (TF paralogs) also enhances the robustness of transcriptional regulatory program [21]. As the two types of TF redundancy have a low overlap, it seems more likely that they work separately. For activity-redundant TFs that share a set of binding target genes, their activity could compensate for each other when one TF is perturbed. On the other hand, TF paralogs tend to have similar binding domains [21]. When robust TFs without activity-redundant TFs are knocked out, their paralogous TFs might be recruited to bind their original target genes to compensate for their loss. As their paralogous TFs have different activity levels from them, the paralogous TFs should be reprogrammed to acquire activity that is similar to theirs. These two types of redundancy guarantee the robustness of transcriptional regulatory program.

We have demonstrated that genes with fragile transcriptional regulatory programs under normal growth condition tend to participate in ESR, though the number of TFs that bind them in stress conditions is comparable with that of robust genes. This implies that the fragility of transcriptional regulatory program under normal growth condition plays an essential role in ESR. Moreover, paralogs also provide backup mechanisms for participation of fragile genes into ESR. As the fragile gene is sensitive to single TF perturbation, the stress-related TFs could readily reprogramme this gene by bypassing only one originally bound TF. In contrast, even though stress-related TFs bind the robust gene, its transcriptional programs might not be altered unless multiple originally bound TFs are bypassed. This makes it difficult for genes with robust transcriptional regulatory programs under normal growth condition to respond to stress conditions. A similar explanation is also applicable to the previous observation that the evolutionary rate of expression or sequence of TFs is not correlated with the *trans *divergence of their target genes [27]. The degree of sensitivity to TF perturbation also affects the contribution of the divergence of TFs on *trans *divergence. Hence, fragile target genes with high divergence of TFs should show high *trans *divergence, whereas robust target genes with high divergence of TFs unnecessarily show high *trans *divergence.

A key finding of this study is that fragile transcriptional regulatory program plays a major role in expression variability. Expression variability also depends on promoter chromatin structure: the presence of nucleosomes close to the TSS is associated with high expression variability [33]. We suggest that the fragility of transcriptional regulatory program is coupled with chromatin structure to determine expression variability. First, the fragile transcriptional regulatory program makes fragile genes sensitive to *trans *variation that could cause expression variability. Second, once the transcriptional regulatory program permits the *trans *variation to act on a gene, the transient removal of nucleosomes that cover TSS and TATA elements is required for gene activation. The fragile transcriptional regulatory program provides a framework that allows for expression variability, and the subsequent eviction of nucleosomes close to the TSS is required for promoter activation.

## Conclusion

**Figure 5:**
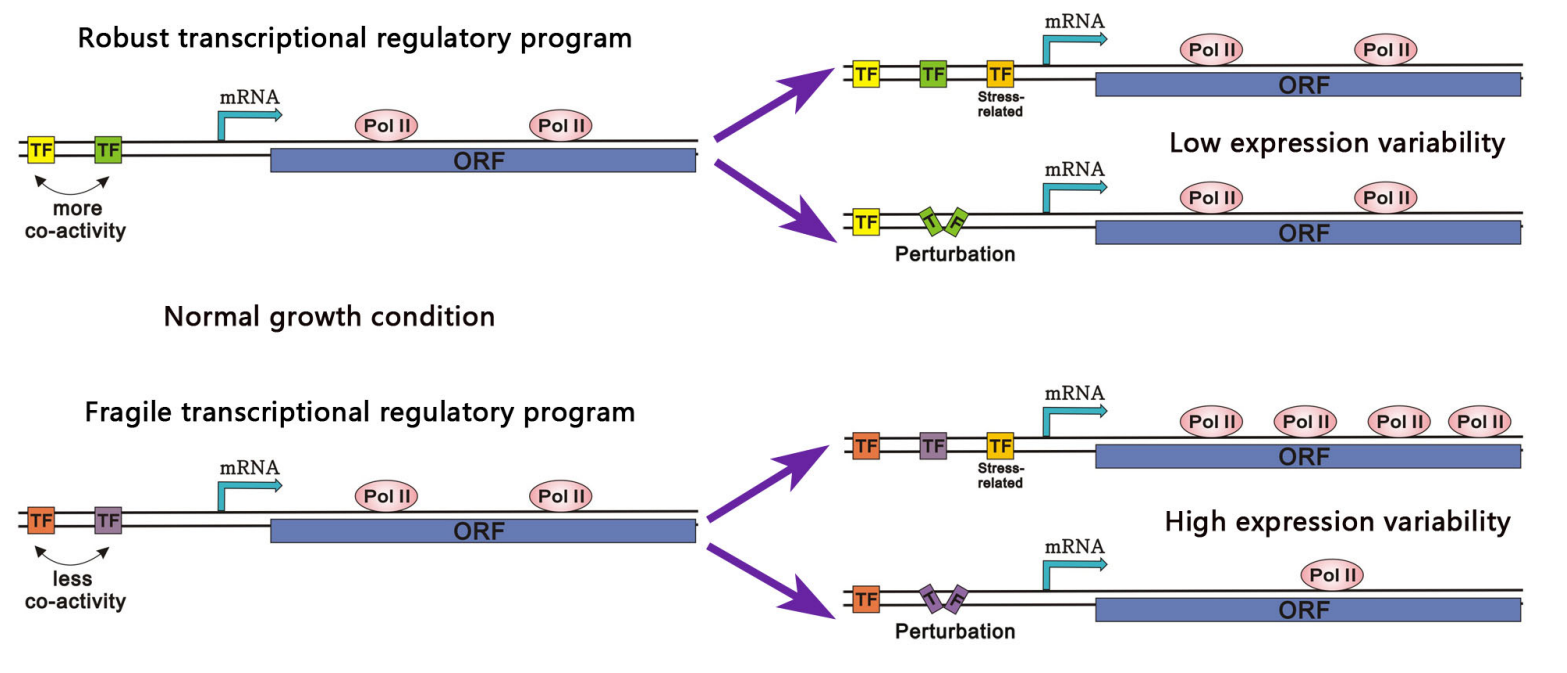
**A model for robust and fragile transcriptional regulatory programs**. Pol II density over the ORF indicates expression level. In the robust transcriptional regulatory program, cooperative TFs work together in an activity-redundant fashion under normal growth condition (the left top panel). Accordingly, the expression level of the robust gene is not affected by stress-related TF binding or TF perturbation (knockout, sequence divergence, activity changes) (the right top two panels). While in the fragile transcriptional regulatory program, cooperative TFs work in an activity-independent fashion under normal growth condition (the left bottom panel). This leads to the expression level of the fragile gene sensitive to stress-related TF binding and TF perturbation (the right bottom two panels).

We have investigated into the cause and effect of robust and fragile transcriptional regulatory programs (Figure 5). In the robust transcriptional regulatory program, cooperative TFs work in concert to regulate their common binding genes in an activity-redundant fashion, and they are able to compensate for each other's loss. Accordingly, their binding genes are insensitive to their changes in activity level and even their single knockout. While in the fragile transcriptional regulatory program, cooperative TFs work together in a relatively activity-independent way, their single perturbation thus significantly affects their binding genes' expression. This high sensitivity to single TF perturbation corresponds to high transcriptional perturbation, which leads to the high expression variability of fragile genes.

## Methods

### TF data

Transcription factor binding data was taken from Harbison et al. [6], which includes the binding affinities of 203 TFs to all promoters under normal growth condition, the binding affinities of 147 TFs to all promoters under various stress conditions. A *P *value cutoff of 0.001 was used to define the set of genes bound by a particular TF. We could determine binding target genes for each TF. Genome-wide changes in expression data corresponding to the knockout of 269 TFs were taken from Hu et al. [5]. As a TF can regulate secondary targets via regulatory cascades, we used their refined transcriptional regulatory network that eliminated indirect regulatory interactions. A *P *value cutoff of 0.001 was used to define the set of genes affected by the knockout of a particular TF. We could determine knockout target genes for each TF. For a given TF, its binding overlap is defined as the percentage value of its binding target genes that are common to its knockout target genes, and its knockout overlap is defined as the percentage value of its knockout target genes that are common to its binding target genes. In this way, we could calculate the binding overlap and the knockout overlap for specific TFs.

### Gene expression data

Genome-wide gene expression data used for coexpression analysis were measured under normal growth conditions [17-19], a total of 112 time points. We calculated for each TF the pair-wise Pearson correlation coefficients among expression profiles of its binding target genes. We calculated for each robust or fragile TF the pair-wise Pearson correlation coefficients between its expression profile and those of its binding target genes, and also between its expression profile and those of its cooperative TFs. For all the 269 TFs [5], we calculated their pair-wise Pearson correlation coefficients in expression profiles. We used the Mann-Whitney U-test to evaluate whether one TF is significantly more or less co-expressed with the rest of the TFs. 68 TFs are more co-expressed than the other TFs (*P *< 0.01, Mann-Whitney U-test), while 67 TFs are less co-expressed than the other TFs (*P *< 0.01, Mann-Whitney U-test).

We compiled available gene expression data from the Stanford Microarray Database [23], a total of 1,260 published microarray experiments for 6,260 genes in various cellular conditions. For each gene, we calculated the average of the squared expression level from the 1,260 experiments as described in a previous study [29], and defined the normalized resulting value as transcriptional plasticity, which reflected the dynamic extent of its expression level in various conditions. Transcriptional plasticity was also calculated separately for up-regulation (the average of the squared positive expression level) or down-regulation (the average of the squared negative expression level). Sensitivity to TF overexpression, which was similarly defined based on a smaller data set of gene expression levels upon overexpression of 55 TFs [22].

Expression divergence data between yeast species were taken from Tirosh et al. [25], which were normalized, such that their means are zero and standard deviations are one. Data for *cis *and *trans *effects to expression divergence between species were taken from Tirosh et al. [27], which were transformed into absolute values. Seven measures were used to represent expression variability as in a previous study [33], including stochastic noise [34], responsiveness [25], stress response [19], *trans *variability [28], mutational variance [35], interstrain variation [36] and expression divergence [25].

### Other data

The TSS data was taken from David et al. [31]. Genome-wide nucleosome occupancy data in vivo were measured with 1-bp resolution by Kaplan et al. [30]. We calculated for each gene the average nucleosome occupancy within the 150 bp upstream of the TSS. The list of ESR genes was taken from Gasch et al. [19]. The list of paralogs was taken from Ding et al. [24]. The list of TATA-containing genes was taken from Basehoar et al. [32]. The list of TFs with the most similar TF paralogs (*E*-value<E-20) was taken from Gitter et al. [21].

### Identification of robust TFs, fragile TFs and their respective cooperative TFs

Given genome-wide TF binding data [6], TF knockout data [5] and gene expression data [17-19], we used following procedures to identify robust TFs, fragile TFs and their respective cooperative TFs. We focused on the 178 TFs that have both binding and knockout data. First, we identified TFs that indeed regulate their own cohorts. For each TF, the pair-wise Pearson correlation coefficients among expression profiles of its binding cohort genes were calculated. If the TF regulates expression of its target genes, its resulting coefficients should be significantly higher than genome-wide levels. For each TF cohort, we selected 100,000 random gene sets with the same size as the TF cohort, and calculated the pair-wise Pearson correlation coefficients in expression profiles for each random gene set. We defined the p-value of a given cohort as the fraction of the same-sized random sets that had higher average coefficient than that of the given cohort (a lower bound of 10^-5 ^on the significance can be assigned to a given cohort). We identified 112 TFs whose cohort genes have significantly higher pair-wise Pearson correlation coefficients in expression profiles (Bonferroni corrected *P *< 0.01). In each cohort, individual genes showing a lower average expressional correlation to all other gene than the average of the cohort were removed. We restricted our analysis to these 112 TFs.

Second, we identified robust and fragile TFs. If a given TF is involved in robust transcriptional regulatory programs, the expression of its cohort genes should not be affected by any TF knockouts. If a given TF is involved in fragile transcriptional regulatory programs, the expression of its cohort genes should be significantly affected by its knockout. We used the Kolmogorov-Smirnov (K-S) statistical test to measure the discrepancy in the distribution of TF-knockout gene expression values between a given TF cohort and the rest of the genes. Our test generated 70 robust TFs whose cohort genes are not differentially expressed upon any TF knockouts (*P *> 0.05, K-S test), and 13 fragile TFs whose cohort genes are differentially expressed upon its knockout (Bonferroni corrected *P *< 0.01, K-S test) (Additional file 1).

Third, we identified cooperative TFs for robust or fragile TF. Given a TF *A*, if another TF *B *works together with *A*, the *A *cohort should be significantly enriched with the *B *binding target genes. We used the Mann-Whitney U-test to evaluate the difference in the medians of experimentally measured *B *binding affinities between the *A *cohort and the rest of the genes. 62 out of 70 robust TFs, and 12 out of 13 fragile TFs have at least one cooperative TF (Additional file 1), that is, the TF cohort genes are significantly bound by at least one other TF (Bonferroni corrected *P *< 0.01, Mann-Whitney U-test).

### Statistical methods

Given two samples of values, the Mann-Whitney U-test is designed to examine whether they have equal medians. The main advantage of this test against t-test is that it makes no assumption that the samples are from normal distributions. Given two samples of values, the Kolmogorov-Smirnov (K-S) test is designed to examine whether they are from the same continuous distribution. The main advantage of this test is that it makes no assumption on the distributions from which the samples originated. The main advantage of multiple linear regression analysis is that it can simultaneously estimate the influence of all factors (the fragility of transcriptional regulatory program and the average nucleosome occupancy within the 150 bp upstream of the TSS in this study).

## Authors' contributions

ZD and XD analyzed the results and drafted the manuscript, and ZD also designed the study, implemented the algorithms, carried out the experiments. QX and JF participated in the analysis and discussion. All authors read and approved the final manuscript.

## Supplementary Material

Additional file 1 The lists of robust TFs, fragile TFs and their cooperative TFs. Click here for file

Additional file 2 **The difference between robust and fragile transcriptional regulatory programs in TF-target co-expression**. We calculated pair-wise Pearson correlation coefficient in expression profiles between robust TFs or fragile TFs and their binding target genes. Distributions of resulting correlation coefficient values are presented for robust TFs (green) and fragile TFs (red). Higher positive correlation indicates more co-expression. Robust TFs are less co-expressed with their binding target genes than fragile TFs. Click here for file

Additional file 3 The list of ORF names for the fragile genes. Click here for file

Additional file 4 **The sensitivity of fragile genes to TF overexpression**. For each gene, we calculated the average of the squared expression level upon overexpression of various TFs, and defined the resulting value as sensitivity to TF overexpression. Average values that correspond to sensitivity to TF overexpression are shown for fragile genes (red) and all genes (violet). Values were normalized, such that their means are zero and standard deviations are one. Error bars were calculated by bootstrapping. Click here for file

Additional file 5 **The transcriptional plasticity of fragile genes**. Average values that correspond to up-regulated and down-regulated transcriptional plasticity are shown for fragile genes (red) and all genes (violet). Values in each property were normalized, such that their means are zero and standard deviations are one. Error bars were calculated by bootstrapping. Click here for file

Additional file 6 **Relationship between fragility of transcriptional regulatory program and transcriptional plasticity**. The fragility of transcriptional regulatory program of one gene is represented by the number of TF knockouts that significantly (*P *< 0.001) affect its expression. All genes were divided into five groups according to the degree of fragility (the five groups correspond to 0, 1~5, 6~10, 11~15, and > = 16 fragility, respectively), and the average transcriptional plasticity was shown for each group. Error bars were calculated by bootstrapping. Click here for file

Additional file 7 The list of ORF names for genes that are bound by multiple TFs in stress conditions. Click here for file

Additional file 8 **Comparison of transcriptional plasticity between fragile genes and TATA-containing genes**. Comparison of transcriptional plasticity was performed between fragile genes (red) and TATA-containing genes (yellow). Values were normalized, such that their means are zero and standard deviations are one. The comparison was also performed when removing the genes that are shared by fragile genes and TATA-containing genes. Error bars were calculated by bootstrapping. Click here for file
