# Supplementary figures and images for: Robustness of transcriptional regulatory program influences gene expression variability
Source: BMC Genomics. 2009 Dec 2;10:573. doi: 10.1186/1471-2164-10-573 (PMC2792230; doi:10.1186/1471-2164-10-573)

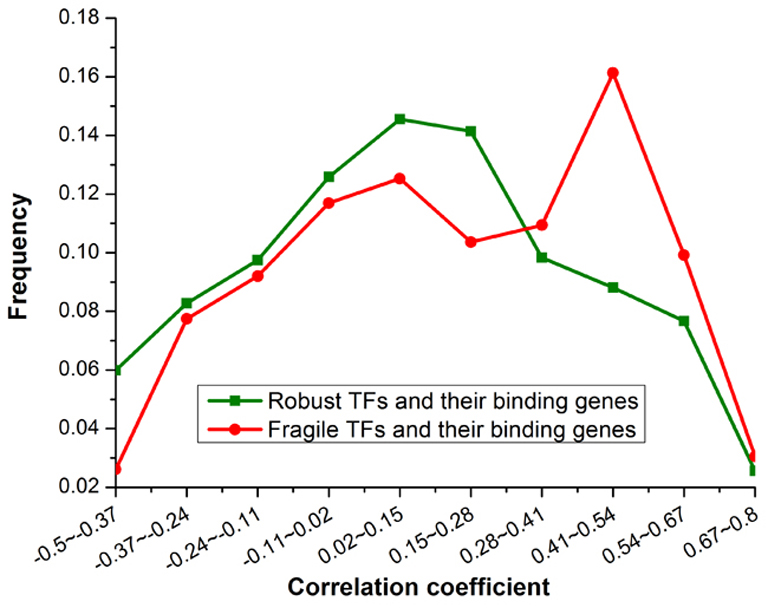

Supplement: Additional file 2 — The difference between robust and fragile transcriptional regulatory programs in TF-target co-expression. We calculated pair-wise Pearson correlation coefficient in expression profiles between robust TFs or fragile TFs and their binding target genes. Distributions of resulting correlation coefficient values are presented for robust TFs (green) and fragile TFs (red). Higher positive correlation indicates more co-expression. Robust TFs are less co-expressed with their binding target genes than fragile TFs. [file 1471-2164-10-573-S2.JPEG]

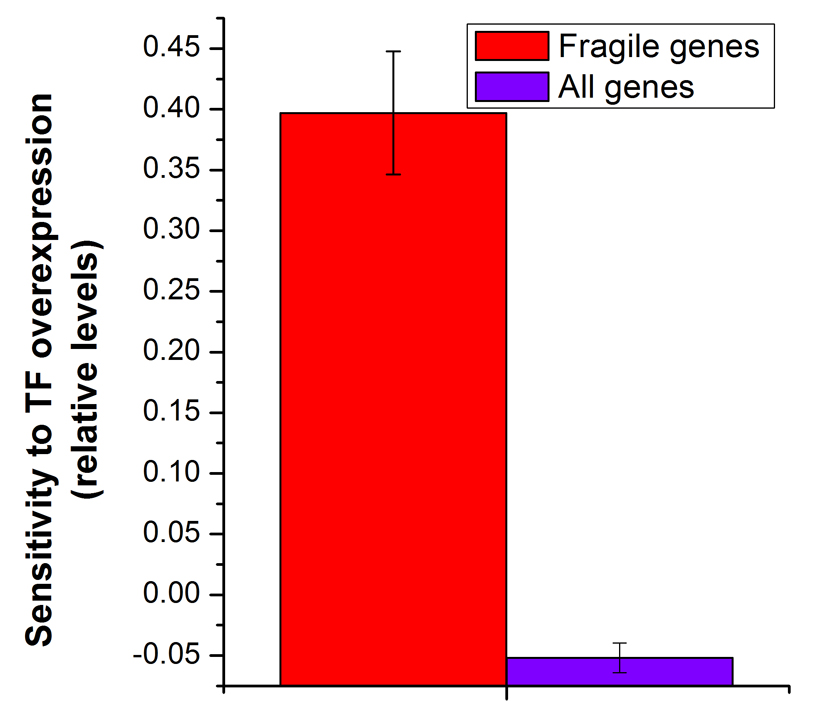

Supplement: Additional file 4 — The sensitivity of fragile genes to TF overexpression. For each gene, we calculated the average of the squared expression level upon overexpression of various TFs, and defined the resulting value as sensitivity to TF overexpression. Average values that correspond to sensitivity to TF overexpression are shown for fragile genes (red) and all genes (violet). Values were normalized, such that their means are zero and standard deviations are one. Error bars were calculated by bootstrapping. [file 1471-2164-10-573-S4.JPEG]

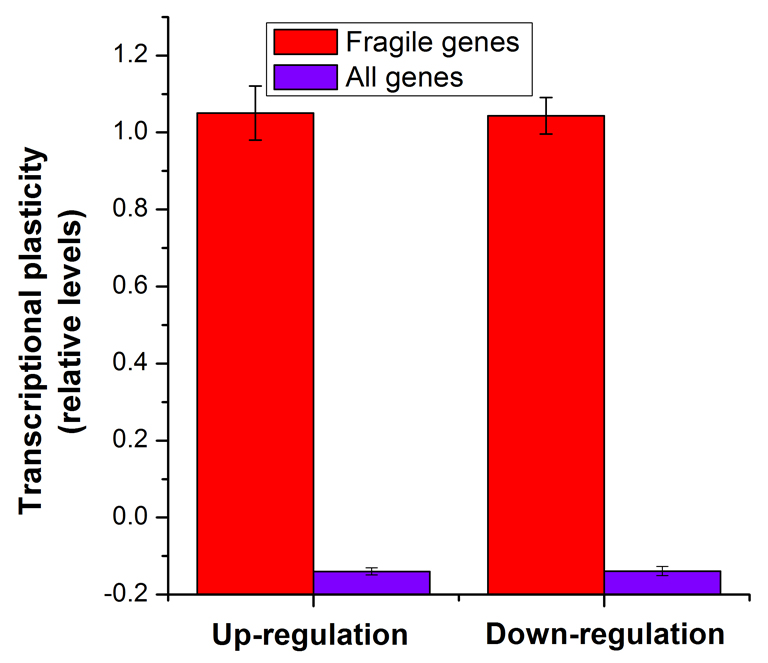

Supplement: Additional file 5 — The transcriptional plasticity of fragile genes. Average values that correspond to up-regulated and down-regulated transcriptional plasticity are shown for fragile genes (red) and all genes (violet). Values in each property were normalized, such that their means are zero and standard deviations are one. Error bars were calculated by bootstrapping. [file 1471-2164-10-573-S5.JPEG]

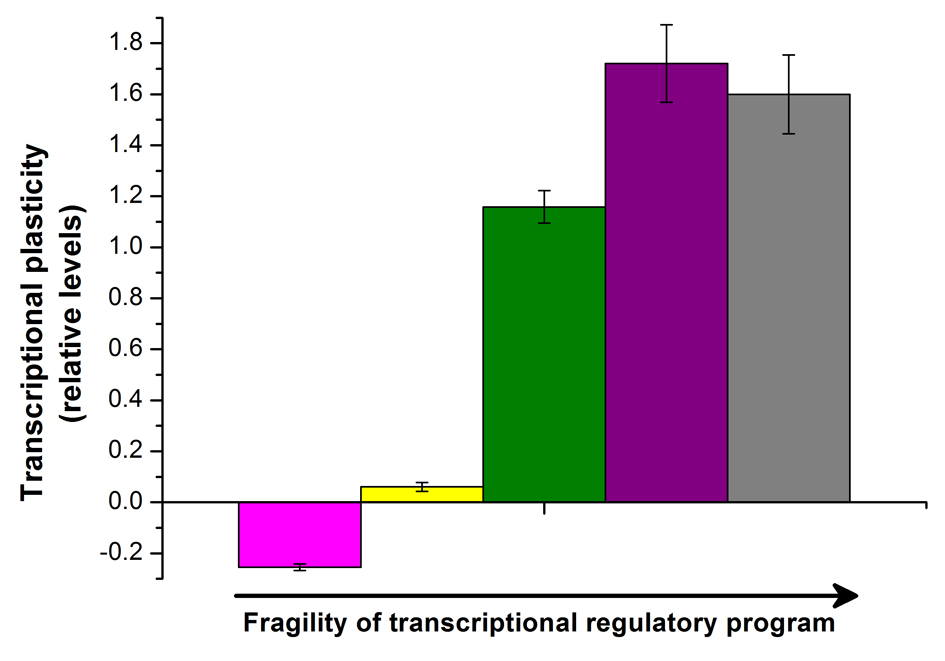

Supplement: Additional file 6 — Relationship between fragility of transcriptional regulatory program and transcriptional plasticity. The fragility of transcriptional regulatory program of one gene is represented by the number of TF knockouts that significantly (P < 0.001) affect its expression. All genes were divided into five groups according to the degree of fragility (the five groups correspond to 0, 1~5, 6~10, 11~15, and > = 16 fragility, respectively), and the average transcriptional plasticity was shown for each group. Error bars were calculated by bootstrapping. [file 1471-2164-10-573-S6.JPEG]

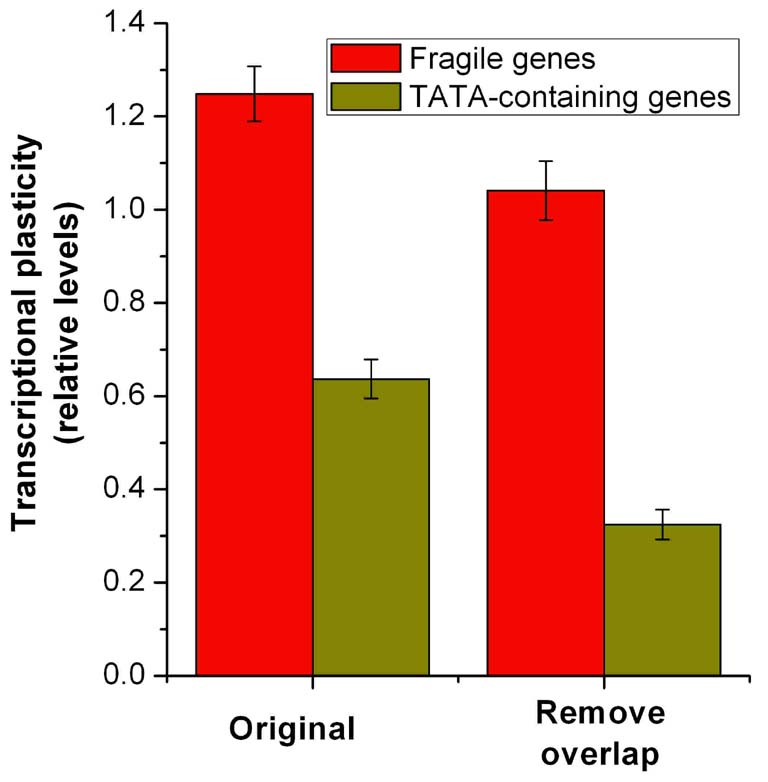

Supplement: Additional file 8 — Comparison of transcriptional plasticity between fragile genes and TATA-containing genes. Comparison of transcriptional plasticity was performed between fragile genes (red) and TATA-containing genes (yellow). Values were normalized, such that their means are zero and standard deviations are one. The comparison was also performed when removing the genes that are shared by fragile genes and TATA-containing genes. Error bars were calculated by bootstrapping. [file 1471-2164-10-573-S8.JPEG]
